# Comparison of three early biomarkers for acute kidney injury after cardiac surgery under cardiopulmonary bypass

**DOI:** 10.1186/s40560-016-0164-1

**Published:** 2016-06-21

**Authors:** Takahiro Moriyama, Shintaro Hagihara, Toko Shiramomo, Misaki Nagaoka, Shohei Iwakawa, Yuichi Kanmura

**Affiliations:** Department of Anesthesiology and Intensive Care, Kagoshima University Hospital, Sakuragaoka 8-35-1, Kagoshima, 46201 Japan; Department of Anesthesiology and Critical Care Medicine, Kyusyu University, Maidashi 3-1-1, Higashi-ku, Fukuoka, 46201 Japan

**Keywords:** Acute kidney injury, Cardiac surgery, L-type fatty acid-binding protein (L-FABP), Neutrophil gelatinase-associated lipocalin (NGAL), Urinary angiotensinogen

## Abstract

**Background:**

Acute kidney injury (AKI) is a serious complication after cardiac surgery, being associated with a high mortality. We assessed three urinary biomarkers, L-type fatty acid-binding protein (L-FABP), neutrophil gelatinase-associated lipocalin (NGAL), and angiotensinogen, which are elevated through different mechanisms, and investigated which of these biomarkers was the earliest and most useful indicator of AKI after cardiac surgery.

**Methods:**

This study was a prospective observational study conducted at a single-institution university hospital. All patients were adults aged under 80 years who underwent cardiac surgery with cardiopulmonary bypass between November 2013 and January 2015. Perioperatively, urine samples were obtained from all patients at five points. Based on AKI criteria, patients were divided into two groups: AKI group (*n* = 11) and non-AKI group (*n* = 39), according to postoperative serum creatinine (Cr) levels.

**Results:**

Urinary L-FABP, NGAL, angiotensinogen, and Cr were measured perioperatively. L-FABP was significantly higher in the AKI group than in the non-AKI group at the end of surgery and 3 h after surgery. L-FABP levels were 601.5 ± 341.7 and 233.8 ± 127.2 μg/g Cr in the AKI and non-AKI groups, respectively. Three hours after surgery, NGAL levels were 950.5 ± 827.9 and 430.0 ± 250.6 μg/g Cr in the AKI and non-AKI groups, respectively, the level being significantly higher in the AKI group than in the non-AKI group. There were no significant differences in urinary angiotensinogen levels between the two groups at any time point.

**Conclusions:**

We demonstrated the utility of L-FABP and NGAL, but not angiotensinogen in the early recognition of AKI. The problem of the different peak points among biomarkers needs to be resolved for discovery of a panel of biomarkers.

## Background

Acute kidney injury (AKI) is one of the most common and serious complications after cardiac surgery, being associated with a high mortality [1]. Depending on the definition used for AKI, its incidence after cardiac surgery reportedly ranges from 5 to 30 % [2, 3]. Traditionally, serum creatinine (sCr) has been the main marker for the diagnosis of AKI. There are three criteria for the diagnosis of AKI, termed the Risk, Injury, Failure, Loss of kidney function and End stage of kidney disease (RIFLE) criteria, Acute Kidney Injury Network (AKIN) criteria, and Kidney Disease: Improving Global Outcomes (KDIGO) criteria [4–6]. These criteria include increasing values and rates of increases of sCr. However, sCr is not an ideal biomarker for AKI after cardiac surgery, because it takes 24–48 h after renal damage for sCr levels to increase, and glomerular filtration rate (GFR) needs to reduce by approximately 75 % before sCr increases to abnormal values [7]. Moreover, previous studies reported that even a small increase in sCr (0 to 0.5 mg/dl) is associated with increasing mortality [3]. sCr also has limited sensitivity and specificity because of several factors such as age, sex, muscle mass, hypertension, and metabolism that can affect its value [8]. Further, renal function can be significantly impaired to an extent that requires renal replacement therapy despite normal sCr levels [9]. A previous study reported that sCr might underestimate the degree of renal failure, especially in elderly patients [10]. Therefore, the importance of earlier and more sensitive biomarkers for the diagnosis of AKI has been recently suggested.

Many urinary and plasma biomarkers for the detection of AKI have been investigated. AKI induced by different mechanisms, such as inflammation, reactive oxygen stress, and intrarenal renin-angiotensin systems, leads to secretion of various biomarkers into urine. In the present study, we assessed three urinary biomarkers, L-type fatty acid-binding protein (L-FABP), neutrophil gelatinase-associated lipocalin (NGAL), and urinary angiotensinogen, whose urinary concentrations are elevated through the different mechanisms. The purpose of this study was to investigate which of these biomarkers is the earliest and most useful indicator of AKI after cardiac surgery under cardiopulmonary bypass (CPB).

## Methods

### Study design and patient population

This prospective observational study was approved by the Ethics Committee of Kagoshima University Hospital and registered with the UMIN Clinical Trials Registry (UMIN 000012312) on November 18, 2013. The present study was conducted in accordance with the principles of the Declaration of Helsinki, and prior written informed consent was obtained from each patient. Fifty patients undergoing cardiac surgery under CPB were enrolled between November 2013 and January 2015. The exclusion criteria for participants in this study were emergency operation, low left ventricular function (ejection fraction <40 %), elderly age (more than 80 years), and preoperative renal dysfunction (sCr level >1.3 mg/dl), all of which might directly increase the risk of occurrence of postoperative AKI.

### General management

Patients were prepared according to the standard preoperative procedures after overnight fasting. Anesthesia was induced by injecting 0.08 mg/kg midazolam, 5 μg/kg fentanyl, and 0.6 mg/kg rocuronium bromide intravenously. Propofol was infused at the rate of 4–10 mg/kg/min to maintain bispectral index (BIS; Aspect Medical Systems, Norwood, MA, USA) within the range of 40–60. Remifentanil was also administered as an infusion, the dose being maintained in the range of 0.1–0.5 μg/kg/min depending on surgical invasiveness. After the induction of anesthesia, the lungs were ventilated to normocapnia and red blood cells were transfused to maintain a hematocrit of 25 % or more.

### Measurements

Urine samples were obtained at five points (before surgery (baseline), before and after CPB, at the end of surgery, and 3 h after surgery). A 5-ml urine sample was collected at each point and stored at −80 °C for later measurements of urinary NGAL, L-FABP, angiotensinogen, and creatinine. Moreover, sCr was measured preoperatively and on postoperative days 1, 2, and 3. Urinary L-FABP, NGAL, and angiotensinogen levels were determined using specific ELISA kits (CMIC Co. Ltd, Tokyo, Bioporto Diagnostics, Hellerup, Denmark, Japan, and IBL, Gunma, Japan, respectively) and were adjusted for urinary creatinine concentrations.

### Definition of AKI

In the present study, we adopted KDIGO criteria to define AKI, according to which AKI after cardiac surgery is defined as an increase in sCr of ≥0.3 mg/dl or a percentage increase of ≥50 % for 48 h after surgery [6]. Based on the KDIGO criteria, patients were divided into two groups: AKI group (*n* = 11) and non-AKI group (*n* = 39).

### Statistical analysis

Data of patient characteristics and clinical outcomes are expressed as mean ± standard deviations (SD). Patient background and outcome variables between the two groups were compared using Fisher’s exact probability test or unpaired Student’s *t* tests using GraphPad Prism version 5. Data of AKI biomarkers are expressed as median ± interquartile range (IQR) and were analyzed with the Wilcoxon test. The performance of urinary biomarkers was determined using receiver operating characteristic (ROC) curve analysis. We considered *P* < 0.05 to be statistically significant.

## Results

A total of 50 patients were divided into two groups based on the KDIGO criteria, with 11 subjects in the AKI group and 39 in the non-AKI group. The patients’ basic and clinical characteristics indicated no significant differences between the two groups, as seen in Table 1. Although there were no differences in sCr concentrations before surgery, sCr was higher in the AKI group than in the non-AKI group at 2 and 3 days after surgery (Table 2). Our results indicated a significantly greater need for renal replacement therapy and longer ICU stay in patients in the AKI group as compared to those in the non-AKI group (Table 3).

**Table 1:** Patient characteristics

	non-AKI group	AKI group
	(*n* = 11)	(*n* = 39)
Age (years)	65.2 ± 11.5	63.8 ± 13.1
Sex (male/female)	4/7	16/23
Body weight (kg)	57.5 ± 13.6	55.1 ± 14.3
Diagnosis		
Coronary artery disease	2	14
Valvular disease	6	20
Ascending aortic aneurysm	3	5
Operating time (min)	384.1 ± 67.4	361.9 ± 69.1
ACC time (min)	128.1 ± 51.4	119.1 ± 57.4
CPB time (min)	162.7 ± 56.9	157.8 ± 52.5
Intraoprerative urine output (ml)	936 ± 168.6	1053 ± 108.6
Intraoperative fluid balance (min)	1978 ± 335.3	2185 ± 196.1

There were no significant differences between the two groups

*ACC* aortic closs-clamp, *CPB* cardio pulmonary bypass

**Table 2:** Pre- and postoperative serum creatinine (sCr) levels

sCr (mg/dl)	AKI group	non-AKI group	*P* value
	(*n* = 11)	(*n* = 39)	
Preoperative	0.72 ± 0.14	0.68 ± 0.15	0.531
Postoperative			
1 day	0.83 ± 0.11	0.69 ± 0.16	<0.05
Postoperative			
2 days	1.04 ± 0.15	0.78 ± 0.19	<0.01
Postoperative			
3 days	1.05 ± 0.12	0.77 ± 0.18	<0.01

**Table 3:** Clinical outcomes

	AKI group	non-AKI group	*P* value
	(*n* = 11)	(*n* = 39)	
AKI severity (*n*)			
Stage 1	6	0	
Stage 2	3	0	
Stage 3	2	0	
Need for RRT (*n*)	3	0	<0.01
ICU stay (day)	8.2 ± 3.5	3.7 ± 2.4	<0.01
ICU death (*n*)	1	0	0.057

*RRT* renal replacement therapy, *ICU* intensive care unit

**Fig. 1:**
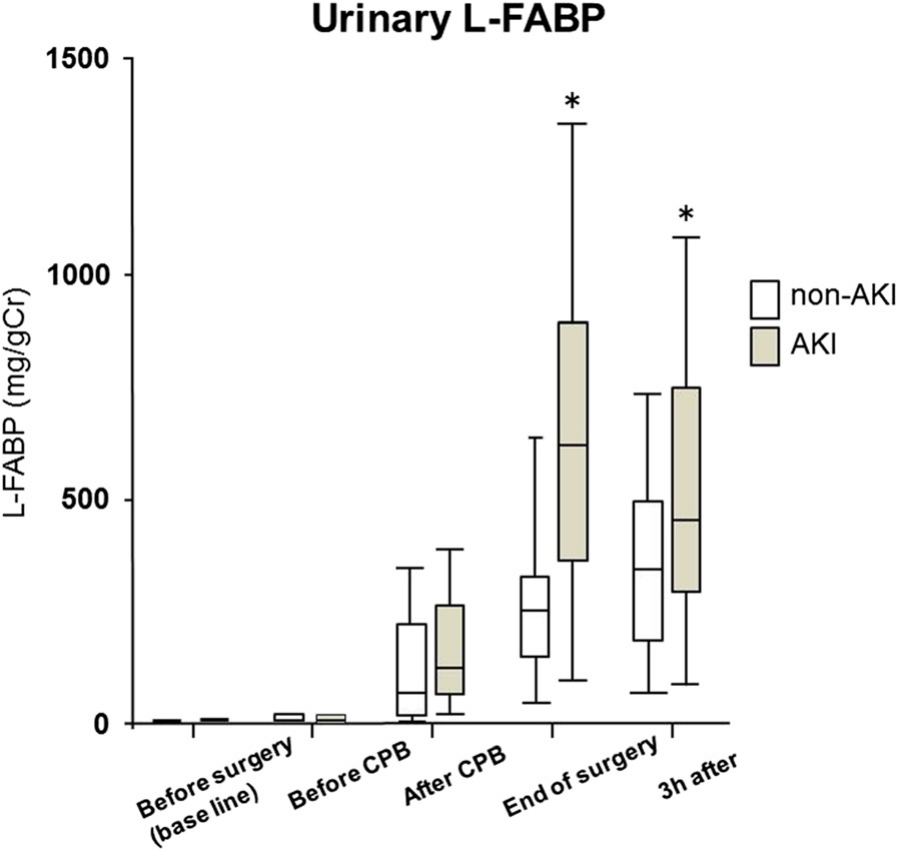
Changes in urinary L-FABP from baseline (before surgery) to 3 h after surgery. Urinary L-FABP levels were significantly higher in the AKI group compared to the non-AKI group at the end of surgery and 3 h after surgery. *P < 0.05. *L-FABP* L-type fatty acid-binding protein, *AKI* acute kidney injury, *CPB* cardiopulmonary bypass

**Fig. 2:**
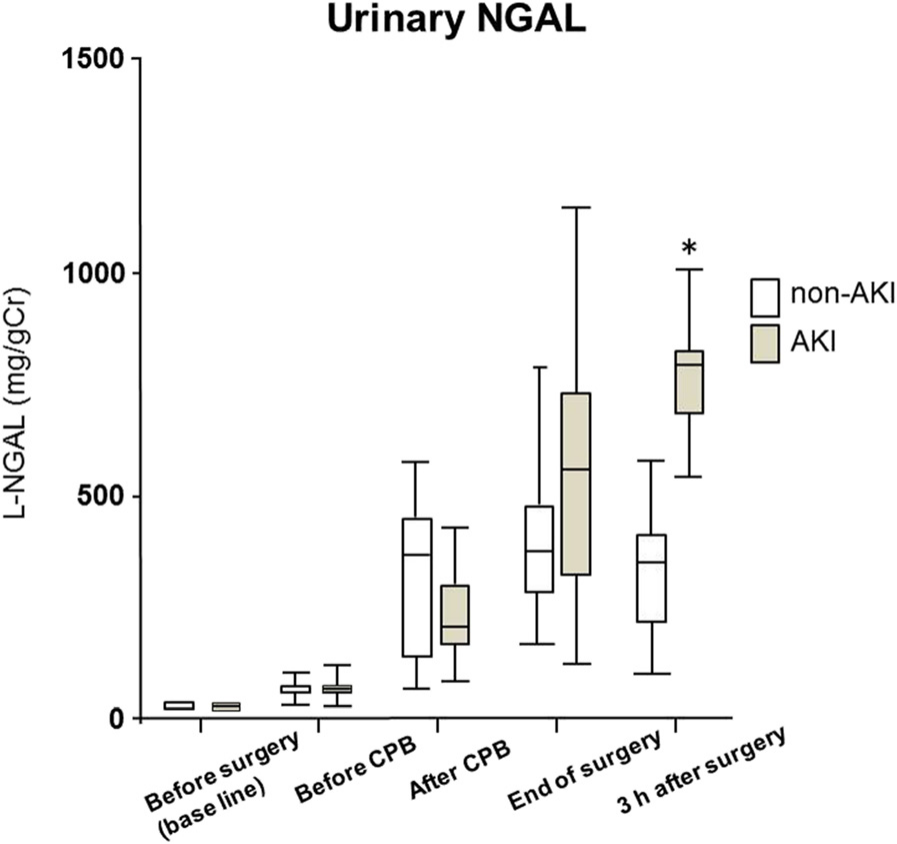
Changes in urinary NGAL from baseline (before surgery) to 3 h after surgery. Urinary NGAL levels were significantly higher in the AKI group compared to the non-AKI group 3 h after surgery. *P < 0.05. *NGAL* neutrophil gelatinase-associated lipocalin, *AKI* acute kidney injury, *CPB* cardiopulmonary bypass

**Fig. 3:**
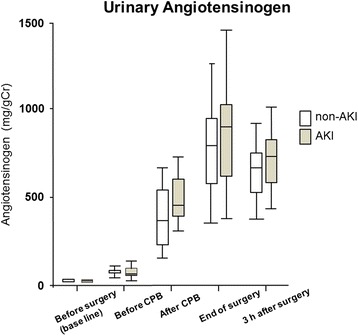
Changes in urinary angiotensinogen from baseline (before surgery) to 3 h after surgery. There were significant differences between the AKI group and non-AKI group. *Ang* angiotensinogen, *AKI* acute kidney injury, *CPB* cardiopulmonary bypass

Urinary L-FABP, NGAL, and angiotensinogen were measured at five time points perioperatively. Each marker increased significantly after CPB compared to baseline levels (before surgery). L-FABP was significantly higher in the AKI group than in the non-AKI group at the end of surgery and 3 h after surgery. L-FABP levels were 601.5 ± 341.7 and 233.8 ± 127.2 μg/g Cr in the AKI and non-AKI groups, respectively, at the end of surgery (Fig. 1). Three hours after surgery, NGAL levels were 950.5 ± 827.9 and 430.0 ± 250.6 μg/g Cr in the AKI and non-AKI groups, respectively, the level being significantly higher in the AKI group than in the non-AKI group for the first time at this point (Fig. 2). In both groups, urinary angiotensinogen levels were the highest at the end of surgery, being 945.3 ± 522.7 and 831.0 ± 419.4 μg/g Cr in the AKI and non-AKI groups, respectively. There were no significant differences in urinary angiotensinogen levels between the two groups at any time point (Fig. 3).

### ROC analysis of urinary L-FABP and NGAL for prediction of AKI

**Fig. 4:**
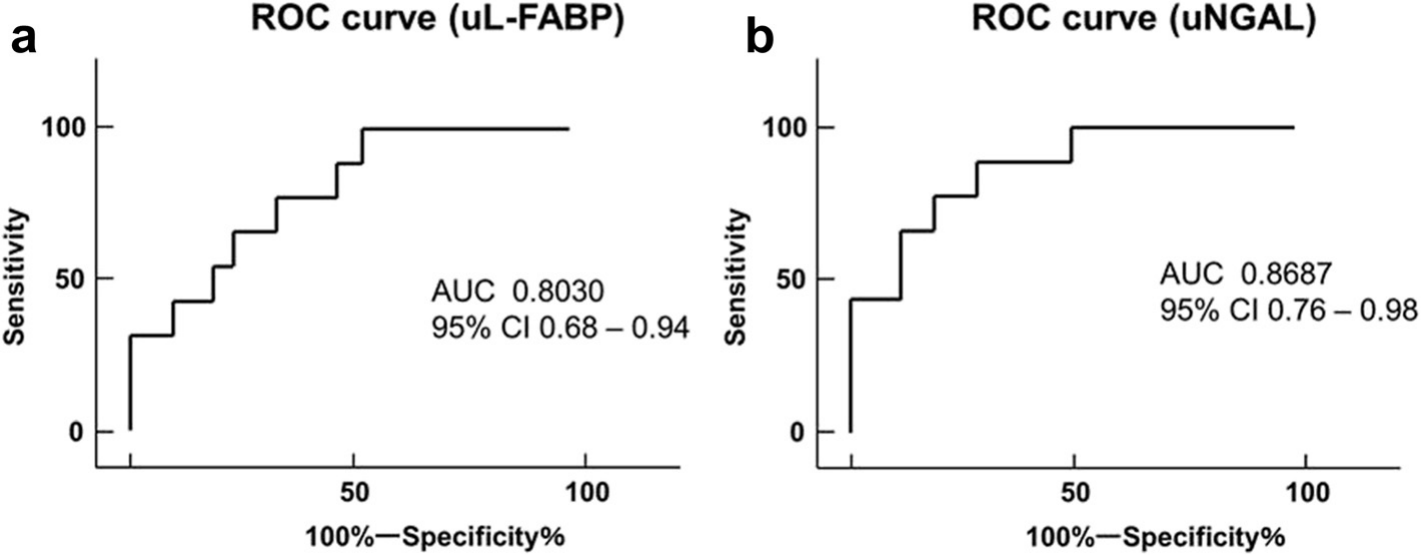
**a** ROC curves for AKI based on uL-FABP levels at the end of surgery. The AUC was 0.8030 (95 % CI 0.68–0.94. *ROC* receiver operating characteristic, *AUC* area under the curve, *uL-FABP* urinary L-type fatty acid-binding protein, *AKI* acute kidney injury. **b** ROC curves for AKI based on uNGAL levels 3 h after surgery. The AUC was 0.8687 (95 % CI 0.76–0.98). *ROC* receiver operating characteristic, *AUC* area under the curve, *uNGAL* urinary neutrophil gelatinase-associated lipocalin, *AKI* acute kidney injury

ROC analysis of L-FABP for the prediction of AKI is shown in Fig. 4a, while Fig. 4b shows ROC analysis of NGAL. These results between the AKI and non-AKI group were analyzed at the most significantly different time points, which were at the end of surgery for L-FABP levels and 3 h after surgery for NGAL levels. The area under the curve (AUC) of NGAL was more than that of L-FABP, although both NGAL and L-FABP were found to be significantly positive predictive markers of AKI. In addition, the cutoff values of L-FABP at the end of surgery and NGAL at 3 h after surgery for diagnosing AKI, as analyzed from ROC curves, were 357.5 and 670.5 μg/g Cr, respectively.

## Discussion

### Key results

In the present study, of the three biomarkers assessed, L-FABP increased most rapidly in the AKI as compared to the non-AKI group. Moreover, there were significant differences between the AKI and non-AKI group in the levels of L-FABP and NGAL. NGAL was a more efficient marker for prediction of the incidence of AKI, because the AUC-ROC of NGAL 3 h after surgery was higher than that of L-FABP at the end of surgery.

### Issues with sCr and the need for new biomarkers for the diagnosis of AKI

sCr has been identified as the main index of kidney injury, and the diagnosis of AKI is mainly based on sCr concentrations in the RIFLE, AKIN, and KDIGO criteria [4–6]. Recently, several research groups reported that small increments in sCr after cardiac surgery, which was not adopted as a diagnostic criterion for AKI, were significantly associated with the progression of postoperative AKI and mortality [3, 11–14]. The underlying mechanism by which a minimal increase in sCr can indicate worsening progression after surgery is not clear. Further, hemodilution and hydration may lead to falsely low sCr measurements, which in turn can confound the diagnosis of AKI after cardiac surgery. This low accuracy of sCr measurements, in addition to the delay in the increase in sCr after cardiac surgery, is another drawback of using sCr as a marker of AKI. With the early detection of AKI, clinical interventions, such as maintaining adequate renal blood flow and blood pressure, and avoiding the development of hypovolemia, anemia, and unnecessary blood transfusion, can be undertaken, which might contribute to reducing the severity of the renal damage.

Therefore, a rapid and efficient biomarker is much needed for the early diagnosis of postoperative AKI. Until now, there was no ideal and complete biomarker, although numerous biomarkers have been previously investigated.

### Urinary L-FABP, NGAL, and angiotensinogen

In the present study, we investigated three urinary biomarkers, L-FABP, NGAL, and angiotensinogen, which are produced and secreted into urine via different mechanisms. L-FABP is a 14-kDa fatty acid-binding protein and is a biomarker that is secreted in urine as a result of reactive oxygen stress. The usefulness of L-FABP as a biomarker in the early detection of AKI after cardiac surgery has been previously reported [15, 16]. NGAL is a 25-kDa protein belonging to the lipocalin superfamily, which is secreted by neutrophils and renal tubular cells [17]. Urinary NGAL has also been recognized as an early biomarker of AKI after cardiac surgery [18, 19]. In the present study, although both urinary NGAL and L-FABP concentrations increased immediately after CPB in both the AKI and non-AKI groups, the levels of urinary L-FABP and NGAL peaked at the end of surgery and 3 h after surgery, respectively. At these time points, the cutoff values of L-FABP and NGAL for diagnosing AKI, as analyzed from ROC curves, were 357.5 and 670.5 μg/g Cr, respectively.

There is a difference between the mechanisms of increase in urinary NGAL and L-FABP after CPB. Oxidative stress via renal tissue hypoxia up-regulates L-FABP expression and increases the secretion of L-FABP into urine from damaged proximal tubules [20]. In contrast, inflammation induced by various stresses increases serum NGAL, while impaired renal absorption increases urinary NGAL excretion [21]. Therefore, the increase in urinary NGAL levels takes a longer time as compared with urinary L-FABP levels [22]. On the other hand, urinary angiotensinogen levels were not significantly different between the two groups at all time points. Recently, the existence of independent renin-angiotensin-aldosterone systems (RAAS) in several organs and tissues has been reported. All components of the renal RAAS are produced in the kidney, being completely independent of the systemic RAAS, and contribute to the progression of both acute and chronic kidney diseases [23]. Moreover, urinary angiotensinogen has been identified as an index of the activity of the renal RAAS [24], and a previous study showed that elevated urinary angiotensinogen was associated with adverse outcomes after cardiac surgery in patients with AKI [25]. Therefore, urinary angiotensinogen has been suggested to have utility as a prognostic biomarker of AKI after cardiac surgery, similar to other previous biomarkers, including L-FABP and NGAL. Indeed, urinary angiotensinogen levels were significantly increased in both the AKI and non-AKI groups after CPB, as compared to baseline (before the surgery). Although the intrarenal RAAS is activated by CPB, the efficacy of urinary angiotensinogen as a biomarker might not be significant, because the activated intrarenal RAAS is merely one of many factors causing AKI after cardiac surgery. Although the usefulness of several biomarkers for detecting AKI have been previously reported [16, 26], in this study, we focused on comparisons between three biomarkers at the same time courses that are produced via different mechanisms. The present results, which confirm the increase in L-FABP and NGAL levels with AKI, suggest that one of the major mechanisms causing AKI after cardiopulmonary bypass might be an increase in reactive oxygen stress, followed by activation of an intrarenal inflammatory reaction.

### Limitations

The present study was conducted at a single institution, and data analysis was not blinded although surgery and anesthesia proceeded normally in all cases. Urinary angiotensinogen concentrations had a wider range as compared to L-FABP and NGAL, suggesting that the sample size might have been too small to assess the utility of these biomarkers for the detection of AKI. A panel of biomarkers would, thus, seem to be more useful for the early detection of AKI [27], although we could not demonstrate the advantage of assessing both L-FABP and NGAL, because the time points at which each of these biomarkers peaked were different. This might also be a limitation of our study.

## Conclusions

We investigated the efficacy of three biomarkers of AKI and demonstrated the utility of L-FABP and NGAL, but not angiotensinogen. All three biomarkers increased after CPB, although they peaked at different time points during the observation period. Hence, although evaluating a panel of biomarkers would probably help overcome the delay in the diagnosis of AKI after cardiac surgery, the problem of the different peak points among biomarkers needs to be resolved for discovery of a panel of biomarkers.

## Availability of supporting data

The datasets supporting the conclusions of this article are included within the article and its additional file.

## Abbreviations

AKI: Acute kidney injury
L-FABP: L-type fatty acid-binding protein
NGAL: Neutrophil gelatinase-associated lipocalin
sCr: Serum creatinine
RIFLE: Risk, Injury, Failure, Loss of kidney function and End stage of kidney disease
AKIN: Acute Kidney Injury Network
GFR: Gomerular filtration rate
CPB: Cardiopulmonary bypass
BIS: Bispectral index
KDIGO: Kidney Disease: Improving Global Outcomes
SD: Standard deviations
ROC: Receiver operating characteristic
RAAS: Renin-angiotensin-aldosterone systems
